# Supplementary material for: Heparin-based hydrogel scaffolding alters the transcriptomic profile and increases the chemoresistance of MDA-MB-231 triple-negative breast cancer cells
Source: Biomater Sci. 2020 Feb 13;8(10):2786–96. doi: 10.1039/c9bm01481k (PMC7497406; doi:10.1039/c9bm01481k)
Supplement: Supplementary file 2 [file BM-008-C9BM01481K-s002.zip › Supplementary File 4/HP-B hydrogel + EGF (s)vControl/Pathways/my_analysis.Gsea.1545201170987/HALLMARK_TNFA_SIGNALING_VIA_NFKB.html]

Details for gene set HALLMARK\_TNFA\_SIGNALING\_VIA\_NFKB[GSEA]

|  || Dataset | expr.class.cls#HYDEGF\_versus\_CONTROL.class.cls#HYDEGF\_versus\_CONTROL\_repos |
| Phenotype | class.cls#HYDEGF\_versus\_CONTROL\_repos |
| Upregulated in class | CONTROL |
| GeneSet | HALLMARK\_TNFA\_SIGNALING\_VIA\_NFKB |
| Enrichment Score (ES) | -0.38366622 |
| Normalized Enrichment Score (NES) | -1.8173795 |
| Nominal p-value | 0.0 |
| FDR q-value | 0.0011519607 |
| FWER p-Value | 0.011 |
Table: GSEA Results Summary

  

Fig 1: Enrichment plot: HALLMARK\_TNFA\_SIGNALING\_VIA\_NFKB      
 Profile of the Running ES Score & Positions of GeneSet Members on the Rank Ordered List

  

| PROBE | DESCRIPTION (from dataset) | GENE SYMBOL | GENE\_TITLE | RANK IN GENE LIST | RANK METRIC SCORE | RUNNING ES | CORE ENRICHMENT || 1 | FOSB | na |  |  | 0 | 4.410 | 0.0258 | No |
| 2 | FJX1 | na |  |  | 71 | 3.118 | 0.0403 | No |
| 3 | EDN1 | na |  |  | 166 | 2.681 | 0.0511 | No |
| 4 | ATF3 | na |  |  | 473 | 2.154 | 0.0476 | No |
| 5 | PTGER4 | na |  |  | 511 | 2.105 | 0.0579 | No |
| 6 | GADD45B | na |  |  | 524 | 2.096 | 0.0695 | No |
| 7 | CCNL1 | na |  |  | 577 | 2.037 | 0.0787 | No |
| 8 | TNFSF9 | na |  |  | 738 | 1.875 | 0.0813 | No |
| 9 | PPP1R15A | na |  |  | 1051 | 1.653 | 0.0745 | No |
| 10 | IER5 | na |  |  | 1276 | 1.523 | 0.0717 | No |
| 11 | EHD1 | na |  |  | 1297 | 1.516 | 0.0795 | No |
| 12 | ID2 | na |  |  | 1377 | 1.479 | 0.0840 | No |
| 13 | PMEPA1 | na |  |  | 1964 | 1.277 | 0.0606 | No |
| 14 | NR4A1 | na |  |  | 1977 | 1.273 | 0.0674 | No |
| 15 | MAP2K3 | na |  |  | 2199 | 1.211 | 0.0629 | No |
| 16 | FOS | na |  |  | 2215 | 1.207 | 0.0692 | No |
| 17 | PLK2 | na |  |  | 2245 | 1.197 | 0.0746 | No |
| 18 | FOSL1 | na |  |  | 2466 | 1.135 | 0.0697 | No |
| 19 | NR4A3 | na |  |  | 2468 | 1.135 | 0.0763 | No |
| 20 | CCND1 | na |  |  | 2684 | 1.084 | 0.0713 | No |
| 21 | GADD45A | na |  |  | 2798 | 1.052 | 0.0715 | No |
| 22 | F3 | na |  |  | 2957 | 1.016 | 0.0692 | No |
| 23 | NFAT5 | na |  |  | 2962 | 1.014 | 0.0749 | No |
| 24 | IRS2 | na |  |  | 3062 | 0.988 | 0.0755 | No |
| 25 | DUSP1 | na |  |  | 3230 | 0.954 | 0.0723 | No |
| 26 | SIK1 | na |  |  | 3781 | 0.850 | 0.0483 | No |
| 27 | MYC | na |  |  | 4314 | 0.764 | 0.0248 | No |
| 28 | TNIP2 | na |  |  | 4345 | 0.759 | 0.0277 | No |
| 29 | MAFF | na |  |  | 4719 | 0.703 | 0.0122 | No |
| 30 | TIPARP | na |  |  | 4770 | 0.692 | 0.0136 | No |
| 31 | SLC2A6 | na |  |  | 4892 | 0.669 | 0.0111 | No |
| 32 | PER1 | na |  |  | 5010 | 0.651 | 0.0088 | No |
| 33 | LIF | na |  |  | 5147 | 0.629 | 0.0053 | No |
| 34 | CD44 | na |  |  | 5293 | 0.607 | 0.0012 | No |
| 35 | STAT5A | na |  |  | 5401 | 0.590 | -0.0009 | No |
| 36 | HES1 | na |  |  | 5622 | 0.558 | -0.0092 | No |
| 37 | JUN | na |  |  | 5642 | 0.555 | -0.0070 | No |
| 38 | PLAU | na |  |  | 5696 | 0.547 | -0.0066 | No |
| 39 | FUT4 | na |  |  | 5981 | 0.505 | -0.0186 | No |
| 40 | SMAD3 | na |  |  | 6056 | 0.496 | -0.0195 | No |
| 41 | MAP3K8 | na |  |  | 6069 | 0.493 | -0.0173 | No |
| 42 | DENND5A | na |  |  | 6175 | 0.479 | -0.0200 | No |
| 43 | SPHK1 | na |  |  | 6182 | 0.478 | -0.0175 | No |
| 44 | TRIP10 | na |  |  | 6198 | 0.476 | -0.0155 | No |
| 45 | IL7R | na |  |  | 6375 | 0.455 | -0.0221 | No |
| 46 | KLF2 | na |  |  | 6417 | 0.447 | -0.0217 | No |
| 47 | CLCF1 | na |  |  | 7212 | 0.342 | -0.0614 | No |
| 48 | KDM6B | na |  |  | 7266 | 0.334 | -0.0622 | No |
| 49 | IL18 | na |  |  | 7482 | 0.306 | -0.0718 | No |
| 50 | ETS2 | na |  |  | 7527 | 0.301 | -0.0723 | No |
| 51 | EGR3 | na |  |  | 7749 | 0.271 | -0.0823 | No |
| 52 | KLF4 | na |  |  | 7914 | 0.254 | -0.0895 | No |
| 53 | LDLR | na |  |  | 8020 | 0.241 | -0.0936 | No |
| 54 | SGK1 | na |  |  | 8039 | 0.239 | -0.0931 | No |
| 55 | NFKB1 | na |  |  | 8333 | 0.205 | -0.1073 | No |
| 56 | RCAN1 | na |  |  | 8703 | 0.163 | -0.1258 | No |
| 57 | BIRC2 | na |  |  | 9633 | 0.057 | -0.1743 | No |
| 58 | IFIT2 | na |  |  | 9862 | 0.027 | -0.1861 | No |
| 59 | SERPINB2 | na |  |  | 9903 | 0.024 | -0.1881 | No |
| 60 | MCL1 | na |  |  | 10007 | 0.019 | -0.1934 | No |
| 61 | IFIH1 | na |  |  | 10011 | 0.019 | -0.1934 | No |
| 62 | LAMB3 | na |  |  | 10015 | 0.019 | -0.1935 | No |
| 63 | EGR1 | na |  |  | 10079 | 0.009 | -0.1968 | No |
| 64 | YRDC | na |  |  | 10328 | -0.026 | -0.2096 | No |
| 65 | HBEGF | na |  |  | 10340 | -0.028 | -0.2101 | No |
| 66 | CEBPB | na |  |  | 10406 | -0.036 | -0.2133 | No |
| 67 | IL15RA | na |  |  | 10423 | -0.038 | -0.2139 | No |
| 68 | TUBB2A | na |  |  | 10631 | -0.057 | -0.2244 | No |
| 69 | CD83 | na |  |  | 10860 | -0.087 | -0.2359 | No |
| 70 | TLR2 | na |  |  | 10870 | -0.089 | -0.2359 | No |
| 71 | IRF1 | na |  |  | 11060 | -0.115 | -0.2451 | No |
| 72 | IER2 | na |  |  | 11245 | -0.129 | -0.2540 | No |
| 73 | RIPK2 | na |  |  | 11399 | -0.146 | -0.2612 | No |
| 74 | RHOB | na |  |  | 11865 | -0.214 | -0.2844 | No |
| 75 | FOSL2 | na |  |  | 12155 | -0.238 | -0.2982 | No |
| 76 | RELB | na |  |  | 12163 | -0.239 | -0.2972 | No |
| 77 | CFLAR | na |  |  | 12192 | -0.242 | -0.2973 | No |
| 78 | RNF19B | na |  |  | 12235 | -0.251 | -0.2980 | No |
| 79 | SOCS3 | na |  |  | 12654 | -0.306 | -0.3182 | No |
| 80 | TGIF1 | na |  |  | 13124 | -0.357 | -0.3408 | No |
| 81 | TNC | na |  |  | 13218 | -0.374 | -0.3435 | No |
| 82 | NINJ1 | na |  |  | 13435 | -0.408 | -0.3524 | No |
| 83 | MXD1 | na |  |  | 13508 | -0.414 | -0.3538 | No |
| 84 | NFKB2 | na |  |  | 13528 | -0.418 | -0.3524 | No |
| 85 | KLF6 | na |  |  | 13608 | -0.426 | -0.3540 | No |
| 86 | SNN | na |  |  | 13639 | -0.432 | -0.3531 | No |
| 87 | NFKBIE | na |  |  | 13693 | -0.440 | -0.3533 | No |
| 88 | ZFP36 | na |  |  | 13904 | -0.472 | -0.3616 | No |
| 89 | SQSTM1 | na |  |  | 13980 | -0.486 | -0.3627 | No |
| 90 | BTG2 | na |  |  | 14115 | -0.493 | -0.3668 | No |
| 91 | RELA | na |  |  | 14142 | -0.496 | -0.3653 | No |
| 92 | PLAUR | na |  |  | 14166 | -0.503 | -0.3636 | No |
| 93 | G0S2 | na |  |  | 14238 | -0.511 | -0.3643 | No |
| 94 | CCL2 | na |  |  | 14503 | -0.557 | -0.3749 | No |
| 95 | INHBA | na |  |  | 14670 | -0.585 | -0.3802 | Yes |
| 96 | BTG3 | na |  |  | 14693 | -0.589 | -0.3780 | Yes |
| 97 | GCH1 | na |  |  | 14797 | -0.603 | -0.3799 | Yes |
| 98 | TNF | na |  |  | 14846 | -0.611 | -0.3788 | Yes |
| 99 | PTPRE | na |  |  | 14865 | -0.614 | -0.3762 | Yes |
| 100 | DNAJB4 | na |  |  | 14878 | -0.616 | -0.3732 | Yes |
| 101 | CDKN1A | na |  |  | 14882 | -0.616 | -0.3697 | Yes |
| 102 | CXCL10 | na |  |  | 14952 | -0.628 | -0.3697 | Yes |
| 103 | REL | na |  |  | 14989 | -0.631 | -0.3679 | Yes |
| 104 | ATP2B1 | na |  |  | 15051 | -0.643 | -0.3674 | Yes |
| 105 | PFKFB3 | na |  |  | 15066 | -0.646 | -0.3643 | Yes |
| 106 | EIF1 | na |  |  | 15103 | -0.654 | -0.3624 | Yes |
| 107 | TNFAIP8 | na |  |  | 15172 | -0.664 | -0.3621 | Yes |
| 108 | EGR2 | na |  |  | 15282 | -0.684 | -0.3638 | Yes |
| 109 | TNIP1 | na |  |  | 15329 | -0.692 | -0.3622 | Yes |
| 110 | F2RL1 | na |  |  | 15344 | -0.696 | -0.3589 | Yes |
| 111 | TRIB1 | na |  |  | 15420 | -0.704 | -0.3587 | Yes |
| 112 | MARCKS | na |  |  | 15423 | -0.704 | -0.3547 | Yes |
| 113 | NFE2L2 | na |  |  | 15575 | -0.737 | -0.3583 | Yes |
| 114 | CXCL2 | na |  |  | 15653 | -0.754 | -0.3579 | Yes |
| 115 | PHLDA2 | na |  |  | 15829 | -0.788 | -0.3625 | Yes |
| 116 | NFIL3 | na |  |  | 15878 | -0.797 | -0.3604 | Yes |
| 117 | PDLIM5 | na |  |  | 15884 | -0.799 | -0.3560 | Yes |
| 118 | KLF9 | na |  |  | 15904 | -0.803 | -0.3523 | Yes |
| 119 | JUNB | na |  |  | 15969 | -0.814 | -0.3509 | Yes |
| 120 | TNFRSF9 | na |  |  | 16000 | -0.819 | -0.3477 | Yes |
| 121 | ZC3H12A | na |  |  | 16069 | -0.838 | -0.3464 | Yes |
| 122 | SERPINB8 | na |  |  | 16076 | -0.839 | -0.3418 | Yes |
| 123 | CSF2 | na |  |  | 16092 | -0.842 | -0.3376 | Yes |
| 124 | JAG1 | na |  |  | 16098 | -0.843 | -0.3330 | Yes |
| 125 | NFKBIA | na |  |  | 16276 | -0.892 | -0.3371 | Yes |
| 126 | IL1A | na |  |  | 16315 | -0.902 | -0.3338 | Yes |
| 127 | ICOSLG | na |  |  | 16433 | -0.928 | -0.3345 | Yes |
| 128 | CEBPD | na |  |  | 16551 | -0.957 | -0.3351 | Yes |
| 129 | CXCL1 | na |  |  | 16603 | -0.969 | -0.3321 | Yes |
| 130 | NR4A2 | na |  |  | 16791 | -1.024 | -0.3359 | Yes |
| 131 | BHLHE40 | na |  |  | 16798 | -1.027 | -0.3302 | Yes |
| 132 | PNRC1 | na |  |  | 16832 | -1.038 | -0.3259 | Yes |
| 133 | TNFAIP3 | na |  |  | 16896 | -1.053 | -0.3231 | Yes |
| 134 | KLF10 | na |  |  | 16932 | -1.064 | -0.3187 | Yes |
| 135 | TSC22D1 | na |  |  | 16950 | -1.072 | -0.3133 | Yes |
| 136 | TRAF1 | na |  |  | 16954 | -1.073 | -0.3072 | Yes |
| 137 | TAP1 | na |  |  | 16995 | -1.089 | -0.3029 | Yes |
| 138 | BCL6 | na |  |  | 17079 | -1.109 | -0.3008 | Yes |
| 139 | ICAM1 | na |  |  | 17147 | -1.127 | -0.2977 | Yes |
| 140 | DUSP5 | na |  |  | 17173 | -1.134 | -0.2924 | Yes |
| 141 | PDE4B | na |  |  | 17197 | -1.143 | -0.2870 | Yes |
| 142 | BCL3 | na |  |  | 17353 | -1.200 | -0.2881 | Yes |
| 143 | CXCL3 | na |  |  | 17374 | -1.206 | -0.2821 | Yes |
| 144 | PANX1 | na |  |  | 17396 | -1.213 | -0.2761 | Yes |
| 145 | GEM | na |  |  | 17445 | -1.234 | -0.2714 | Yes |
| 146 | DDX58 | na |  |  | 17467 | -1.244 | -0.2652 | Yes |
| 147 | EFNA1 | na |  |  | 17471 | -1.245 | -0.2581 | Yes |
| 148 | ZBTB10 | na |  |  | 17658 | -1.317 | -0.2602 | Yes |
| 149 | DRAM1 | na |  |  | 17740 | -1.352 | -0.2566 | Yes |
| 150 | BTG1 | na |  |  | 17748 | -1.358 | -0.2490 | Yes |
| 151 | DUSP4 | na |  |  | 17837 | -1.409 | -0.2454 | Yes |
| 152 | PTX3 | na |  |  | 17871 | -1.426 | -0.2388 | Yes |
| 153 | ABCA1 | na |  |  | 17887 | -1.432 | -0.2312 | Yes |
| 154 | PHLDA1 | na |  |  | 17992 | -1.481 | -0.2280 | Yes |
| 155 | PTGS2 | na |  |  | 17994 | -1.483 | -0.2194 | Yes |
| 156 | B4GALT1 | na |  |  | 18015 | -1.493 | -0.2117 | Yes |
| 157 | GFPT2 | na |  |  | 18069 | -1.525 | -0.2056 | Yes |
| 158 | TANK | na |  |  | 18094 | -1.545 | -0.1978 | Yes |
| 159 | LITAF | na |  |  | 18114 | -1.555 | -0.1897 | Yes |
| 160 | IL1B | na |  |  | 18115 | -1.556 | -0.1806 | Yes |
| 161 | IL23A | na |  |  | 18181 | -1.597 | -0.1747 | Yes |
| 162 | IL6 | na |  |  | 18227 | -1.619 | -0.1676 | Yes |
| 163 | B4GALT5 | na |  |  | 18251 | -1.640 | -0.1592 | Yes |
| 164 | SPSB1 | na |  |  | 18303 | -1.685 | -0.1521 | Yes |
| 165 | CCL20 | na |  |  | 18309 | -1.693 | -0.1424 | Yes |
| 166 | IFNGR2 | na |  |  | 18361 | -1.722 | -0.1350 | Yes |
| 167 | IL6ST | na |  |  | 18442 | -1.792 | -0.1288 | Yes |
| 168 | BMP2 | na |  |  | 18629 | -1.962 | -0.1271 | Yes |
| 169 | SDC4 | na |  |  | 18719 | -2.074 | -0.1196 | Yes |
| 170 | AREG | na |  |  | 18856 | -2.288 | -0.1134 | Yes |
| 171 | CCRL2 | na |  |  | 18863 | -2.300 | -0.1003 | Yes |
| 172 | BIRC3 | na |  |  | 18942 | -2.435 | -0.0901 | Yes |
| 173 | NAMPT | na |  |  | 18985 | -2.547 | -0.0774 | Yes |
| 174 | SOD2 | na |  |  | 18995 | -2.569 | -0.0629 | Yes |
| 175 | SLC2A3 | na |  |  | 19022 | -2.648 | -0.0488 | Yes |
| 176 | CXCL11 | na |  |  | 19056 | -2.745 | -0.0345 | Yes |
| 177 | SAT1 | na |  |  | 19134 | -3.131 | -0.0202 | Yes |
| 178 | KYNU | na |  |  | 19189 | -4.024 | 0.0005 | Yes |
Table: GSEA details [plain text format]

  

Fig 2: HALLMARK\_TNFA\_SIGNALING\_VIA\_NFKB      
 Blue-Pink O' Gram in the Space of the Analyzed GeneSet

  

Fig 3: HALLMARK\_TNFA\_SIGNALING\_VIA\_NFKB: Random ES distribution      
 Gene set null distribution of ES for **HALLMARK\_TNFA\_SIGNALING\_VIA\_NFKB**
